# Astaxanthin acts via LRP-1 to inhibit inflammation and reverse lipopolysaccharide-induced M1/M2 polarization of microglial cells

**DOI:** 10.18632/oncotarget.20628

**Published:** 2017-09-03

**Authors:** Xiaojun Wen, Lijiao Xiao, Zhuoyan Zhong, Limin Wang, Ze Li, Xiaoping Pan, Zhonglin Liu

**Affiliations:** ^1^ Department of Neurology, Guangzhou First People’s Hospital, Guangzhou Medical University, The Second Affiliated Hospital, South China University of Technology, Guangzhou, China; ^2^ Department of Neurology, Sun Yat-Sen Memorial Hospital, Sun Yat-Sen University, Guangzhou, China; ^3^ Department of Neurology, Guangdong General Hospital and Guangdong Academy of Medical Sciences, Guangzhou, China; ^4^ Guangdong Provincial Key Laboratory of Malignant Tumor Epigenetics and Gene Regulation, Guangzhou, China

**Keywords:** astaxanthin, M1/M2 phenotypes, low-density lipoprotein receptor-related protein 1, nuclear factor-κB, c-Jun N-terminal kinase, Gerotarget

## Abstract

Microglia become activated during neuroinflammation and produce neurotoxic and neurotrophic factors, depending on whether they acquire M1 proinflammatory or M2 anti-inflammatory phenotypes. Astaxanthin (ATX), a natural carotenoid, has anti-inflammatory and neuroprotective effects. We investigated whether ATX could reverse M1/M2 polarization and suppress neuroinflammation *via* low-density lipoprotein receptor-related protein-1 (LRP-1). We observed increased expression of M1 (TNF-α, IL-1β, and CD86) and decreased expression of M2 (Arg-1, IL-10, and CD206) markers in BV2 microglial cells stimulated with lipopolysaccharide (LPS). These alterations were reversed by pretreating the cells with ATX. Activation of the NF-κB and JNK pathways was observed upon LPS stimulation, which was reversed by ATX. ATX-induced M2 polarization was attenuated by inhibition of NF-κB and JNK. Pretreatment of LPS-stimulated BV2 cells with ATX resulted in increased LRP-1 expression. The addition of receptor-associated protein, an LRP-1 antagonist, ameliorated ATX-induced inactivation of NF-κB and JNK signaling, and M2 polarization. ATX promotes M2 polarization to suppress neuroinflammation *via* LRP-1 by inhibiting NF-κB and JNK signaling. This novel mechanism may suppress neuroinflammation in diseases such as Alzheimer’s disease.

## INTRODUCTION

Microglia-mediated neuroinflammation is a prominent and early feature of Alzheimer’s disease (AD) [1-2]. Polarization of resident microglia is a functional dichotomy involving classical M1 and alternative M2 phenotypes [3]. M1 polarized microglia are characterized by an overproduction of inflammatory cytokines including IL-1 and TNF-α. In contrast, M2 polarized microglia are characterized by the release of anti-inflammatory mediators including IL-10 and TGF-β, which leads to regeneration and neuroprotection [4-5]. Continued polarization towards an M1 phenotype results in inflammation [6]. Therapeutics that promote the differentiation of microglia from an M1 to M2 phenotype could be effective in neuroinflammatory diseases such as AD [4, 7-8].

Low-density lipoprotein receptor-related protein-1 (LRP-1) is an endocytic receptor that is highly expressed on neurons and glial cells [9]. It plays an essential role in the clearance of amyloid-β (Aβ) from the central nervous system [10-11]. Reduced low-density lipoprotein receptor-related protein (LRP) levels have been observed in AD patients compared to healthy controls [12]. Additionally, increased LRP-1 levels in AD patients were correlated with a later age of disease onset, indicating higher LRP levels might be protective against AD. LRP-1 suppressed the lipopolysaccharide (LPS)-induced inflammatory response *via* the interferon-γ promoter in macrophages [13]. Activation of LRP-1 could modulate microglial inflammation by regulating the JNK and NF-κB signaling pathways [14-15]. LRP-1 deficiency resulted in down-regulation of M2 marker expression in macrophages and promoted M1 polarization [16]. Thus, LRP-1 may protect against inflammation.

Astaxanthin (ATX) is a naturally carotenoid pigment found in many marine organisms [17]. It has anti-inflammatory and anti-carcinogenic activity as well as neuroprotective effects [18-20]. ATX induced down-regulation of IL-6 through an NF-κB p65-dependent pathway in activated BV2 microglial cells [21] and inhibited NF-κB activity by down-regulating IKK [22]. We previously demonstrated that ATX protected glutamate-induced cytotoxicity in HT22 cells by attenuating caspase activation and mitochondrial dysfunction, and modulating Akt/GSK-3β signaling [20].

We investigated the effects of ATX on M1/M2 microglia polarization and inflammation. Pretreatment of LPS-stimulated BV2 cells with ATX promoted an M2 neuroprotective phenotype. We observed an increase in the expression of M2 (Arg-1, IL-10, and CD206) and a decrease in the expression of M1 (TNF-α, IL-1β, and CD86) markers. We found that LRP-1 was a target of ATX and demonstrated that the NF-κB and JNK signaling pathways comprise the core mechanisms underlying ATX-mediated suppression of neuroinflammation.

## RESULTS

### LPS differentially induces M1 and M2 marker expression in BV2 cells

**Figure 1:**
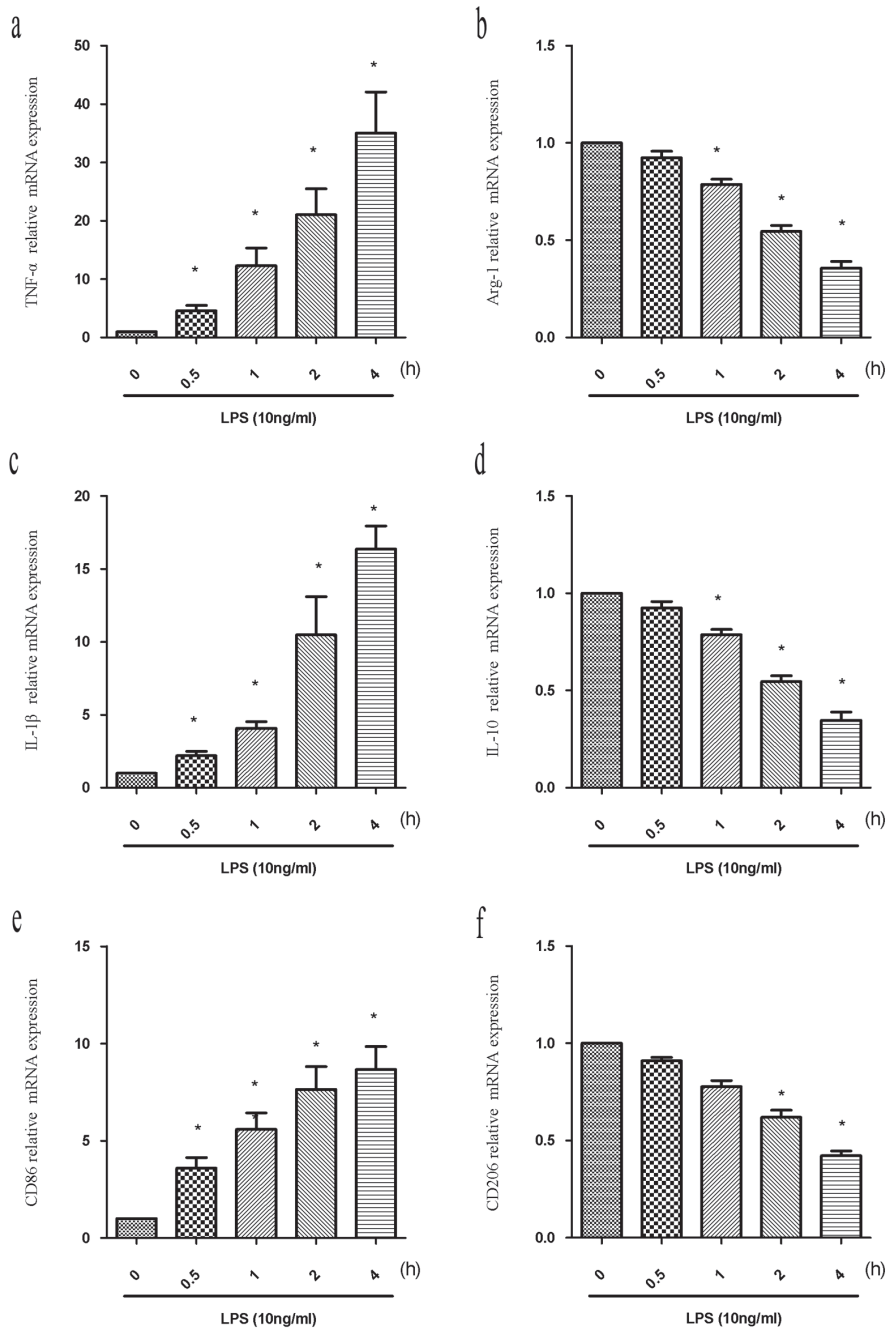
LPS induces alterations in the expression of M1 and M2 markers BV2 cells were treated with LPS (10 ng/mL) for the indicated times (0, 0.5, 1, 2, or 4 h). Total RNA was extracted and the expression of M1 markers (TNF-α, IL-1β, and CD86) and M2 markers (Arg-1, IL-10, and CD206) analyzed by RT-PCR. Fold changes in gene expression were quantified relative to β-actin. **a.**, **c.**, **e.** M1 marker expression. **b.**, **d.**, **f.** M2 marker expression. Data are presented as the mean ± SD (*n* = 3). ^*^P < 0.05 compared to controls.

Reactive polarized microglia are characterized by differential expression of inflammatory cytokines and cell surface markers. To evaluate M1/M2 polarization, we analyzed the expression of M1 and M2 cytokines (TNF-α and IL-1β, and Arg-1 and IL-10, respectively), and surface markers (CD86 and CD206, respectively) in BV2 cells after treatment with LPS (10 ng/mL) for 0, 0.5, 1, 2, or 4 h using quantitative reverse transcription PCR (RT-PCR). We observed a time-dependent increase in M1 marker expression (TNF-α, IL-1β, and CD86) between 0.5 and 4 h in LPS-treated compared to control cells (Figure 1a, 1c, 1e) (*P* < 0.05). In contrast, a time-dependent decrease in the expression of M2 markers was observed between 0.5 and 4 h. Significant decreases in Arg-1 and IL-10 were observed at 1, 2, and 4 h, and in CD206 at 2 h and 4 h compared to untreated control cells (Figure 1b, 1d, 1f) (*P* < 0.05). Thus, LPS induced M1 microglia activation and inhibited M2 polarization in BV2 cells.

### ATX suppresses LPS-induced M1 polarization and promotes M2 polarization in BV2 cells

**Figure 2:**
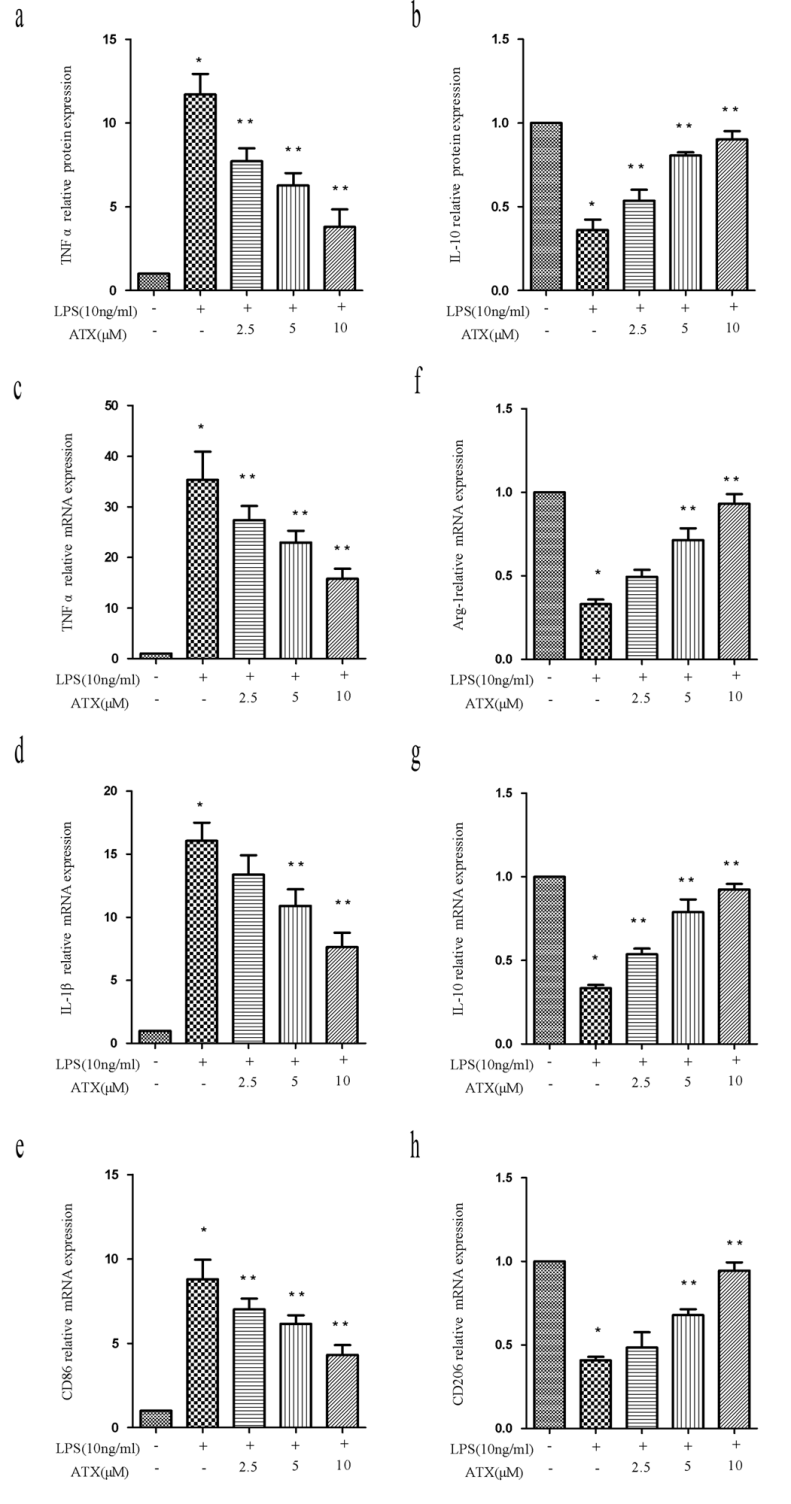
ATX inhibits LPS-induced M1 and promotes M2 polarization in BV2 cells **a.**-**b.** BV2 cells were incubated with ATX at the indicated concentrations (0, 2.5, 5, and 10 μM) for 4 h. Cells were then incubated with LPS (10 ng/mL) for 4 h. TNF-α and IL-10 production in the media was examined using ELISA. **c.**-**h.** BV2 cells were incubated with ATX at the indicated concentrations (0, 2.5, 5, and 10 μM) for 4 h. LPS (10 ng/mL) was then added for 4 h. Total RNA was extracted and the expression of M1 (TNF-α, IL-1β, and CD86) and M2 (Arg-1, IL-10, and CD206) markers analyzed by RT-PCR. Fold changes in gene expression were quantified relative to β-actin. **c.**-**e.** M1 markers. **f.**-**h.** M2 markers. Data are presented as the mean ± SD (*n* = 3). ^*^P < 0.05 indicates a significant difference in the expression of the different markers relative to untreated [LPS (−)/ATX (−)] controls. ^**^P < 0.05 indicates a significant difference in the expression of the different markers compared to LPS (+)/ATX (−)-treated cells.

We investigated the anti-inflammatory effects of ATX at various concentrations (0 to 10 μM) and M1/M2 polarization in LPS-stimulated BV2 cells. ELISA data indicated ATX inhibited LPS-induced TNF-α release from BV2 cells in a dose-dependent manner. The highest inhibition was observed at 10 μM (Figure 2a, *P* < 0.05). Conversely, ATX induced a dose-dependent increase in IL-10 release from BV2 cells (Figure 2b, *P* < 0.05). RT-PCR analysis demonstrated that ATX reduced the expression of M1 markers (TNF-α, IL-1β, and CD86) in a dose-dependent manner (Figure 2c-2e). A significant decrease in TNF-α and CD86 was observed at 2.5 to 10 μM, and in IL-1β between 5 and 10 μM compared to LPS-treated control cells (*P* < 0.05). ATX also induced a dose-dependent increase in the expression of M2 markers (Arg-1, IL-10, and CD206) (Figure 2f-2h). A significant increase in IL-10 was observed between 2.5 and 10 μM (IL-10), and in Arg-1 and CD206 at 5 and 10 μM compared to cells treated with LPS alone (*P* < 0.05). Thus, ATX inhibited LPS-induced M1 microglia activation while promoting M2 polarization in BV2 cells.

### ATX reverses LPS-induced microglia polarization by inhibiting NF-κB and JNK activation

**Figure 3:**
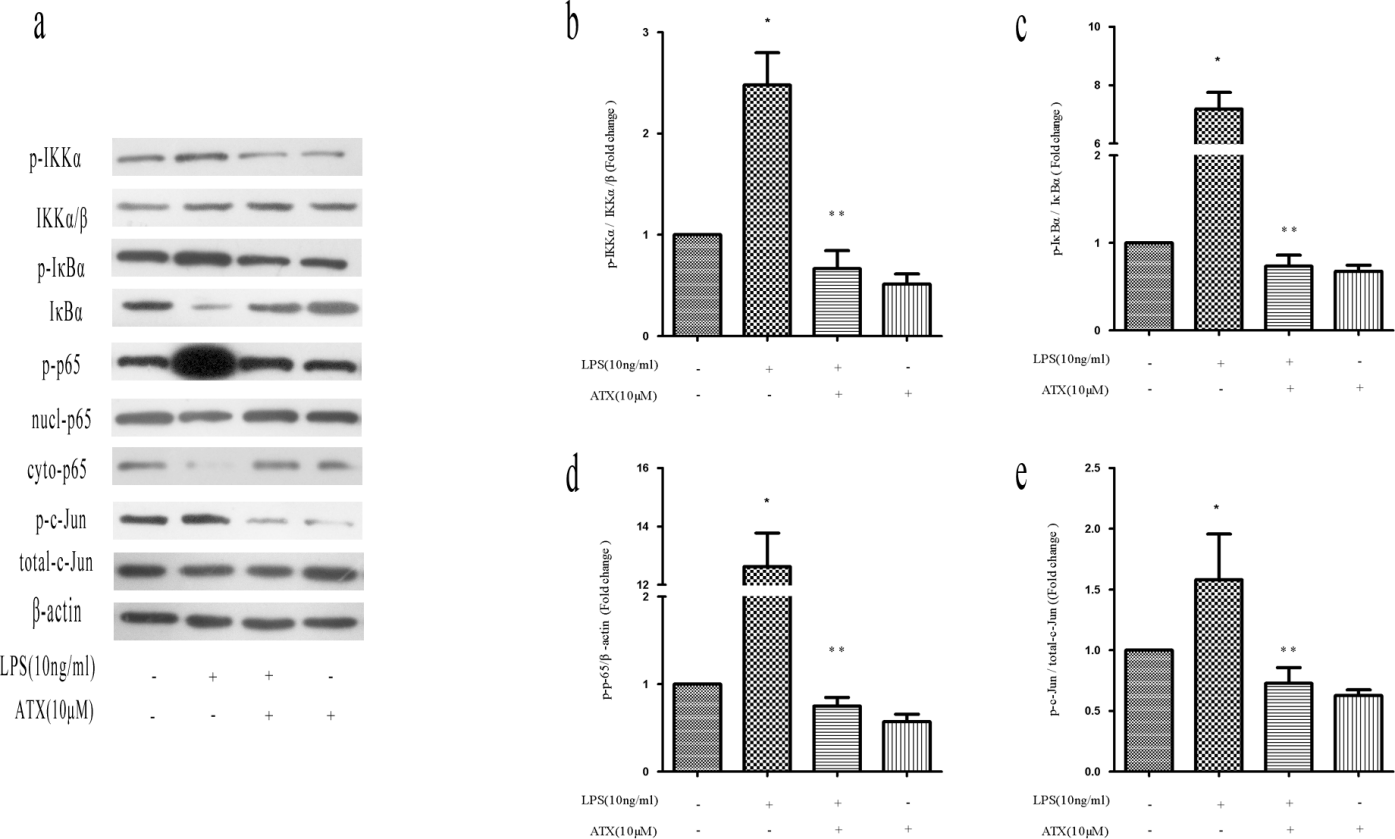
ATX reverses LPS-induced microglia polarization by inhibiting NF-κB and JNK signaling BV2 cells were incubated with ATX at the indicated concentrations (0, 2.5, 5, and 10 μM) for 4 h. LPS (10 ng/mL) was then added for 4 h. **a.** Western blot analysis of NF-κB and JNK pathway proteins. **b.** Analysis of the ratio of p-IKKα/p-IKKα/β expression. **c.** Analysis of the ratio of p-IκBα/IκBα expression. **d.** Analysis of the ratio of p-p65/β-actin expression. **e.** Analysis of the ratio of p-c-Jun/total-c-Jun expression. Data are presented as the mean ± SD (*n* = 3). ^*^P < 0.05 indicates a significant difference in the expression of the different markers relative to untreated [LPS (−)/ATX (−)] controls. ^**^P < 0.05 indicates a significant difference in the expression of the different markers compared to LPS (+)/ATX (−)-treated cells.

To understand the potential mechanisms by which ATX reversed LPS-induced M1 polarization, we analyzed NF-κB and c-Jun N-terminal kinase (JNK) pathway activation, which functions downstream of LPS stimulation [23-24]. We examined the phosphorylation of IKKα, IκBα, and NF-κB p65 by western blotting. Pretreatment of LPS-stimulated BV2 cells with ATX reduced the levels of p-IKKα, p-IκBα, and p-p65. The levels of p-IκBα increased after LPS stimulation whereas the levels of IκBα decreased, resulting in the translocation of NF-κB p65 into the nucleus. These effects were reversed by pretreatment with ATX (Figure 3a). IκBα/β levels did not change after LPS stimulation in the presence or absence of ATX (Figure 3a). Similar effects were observed in band intensity (Figure 3b-3d). Under normal conditions, NF-κB p65 was cytoplasmic. Analysis of nuclear and cytosolic extracts from BV2 cells revealed a decrease in p65 translocation from the cytoplasm to the nucleus after pretreatment with ATX (Figure 3a). In addition, the level of p-c-Jun was higher after LPS stimulation compared to control cells, and was attenuated upon pretreatment with ATX (Figure 3a, 3e). We found that ATX inhibited IKKα, IκBα, p65, and c-Jun phosphorylation in LPS-induced BV2 microglial cells. These results suggested that ATX reversed LPS-induced microglia polarization by inhibiting NF-κB and JNK pathway activation.

### ATX reverses the LPS-induced reduction in LRP-1 expression in BV2 cells

**Figure 4:**
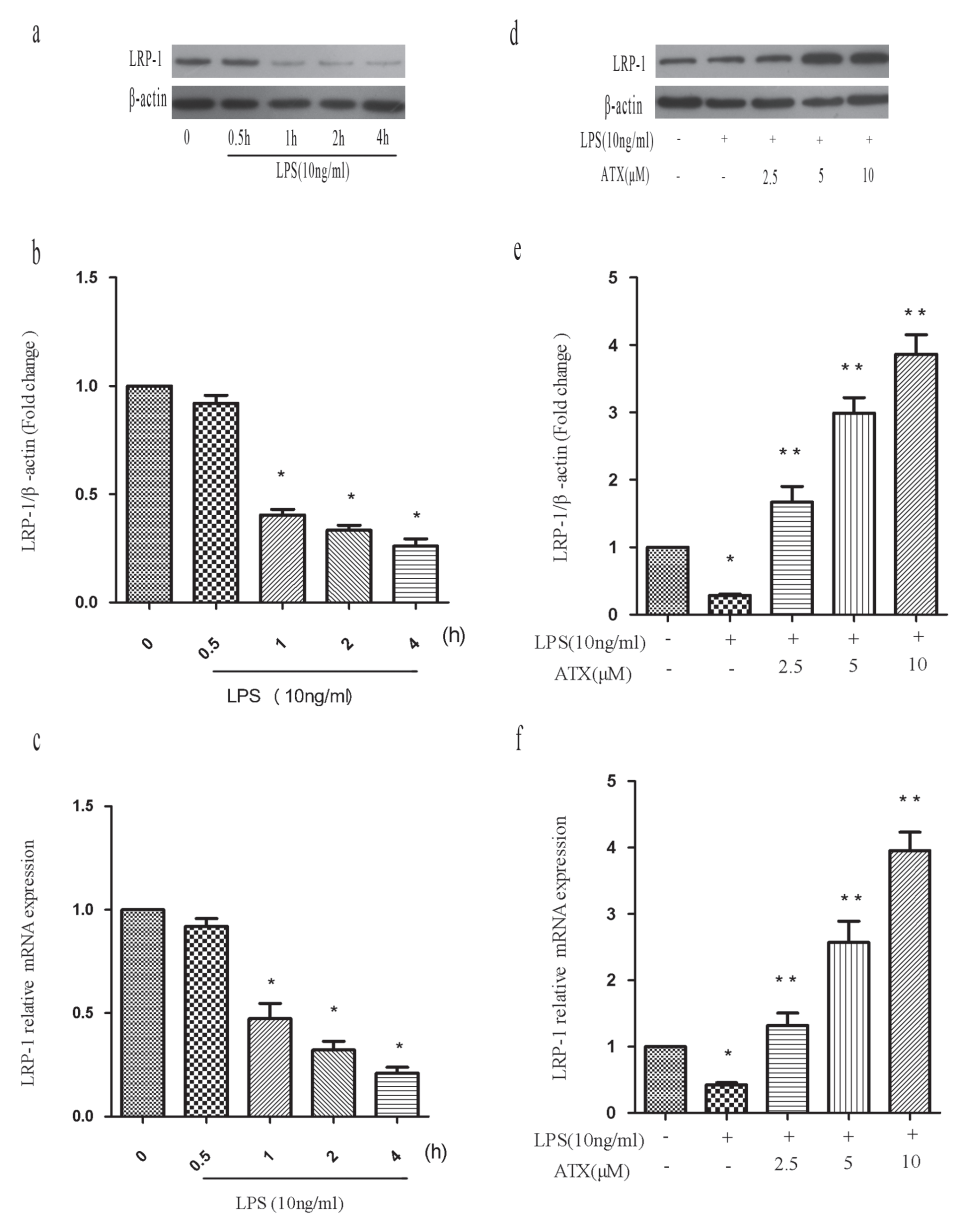
ATX reverses the LPS-induced reduction in LRP-1 expression in BV2 cells **a.**-**c.** BV2 cells were treated with LPS (10 ng/mL) for the indicated times (0, 0.5, 1, 2, and 4 h) and LRP-1 mRNA and protein levels analyzed. Fold changes in LRP-1 expression relative to β-actin are shown. **d**.-**f**. BV2 cells were incubated with ATX at the indicated concentrations (0, 2.5, 5, and 10 μM) for 4 h and then incubated with LPS (10 ng/mL) for 4 h. LRP-1 mRNA and protein levels were analyzed. Data are presented as the mean ± SD (*n* = 3). ^*^P < 0.05 indicates a significant difference in the expression of the different markers relative to untreated [LPS (−)/ATX (−)] controls. ^**^P < 0.05 indicates a significant difference in the expression of the different markers compared to LPS (+)/ATX (−)-treated cells.

Previous studies have found that various stimuli including LPS cause down-regulation of LRP-1 expression, indicating LRP-1 has an essential role in regulating microglia activation [25-26]. Therefore, we analyzed LRP-1 levels in the presence and absence of ATX. We treated BV2 cells with LPS (10 ng/mL) for the indicated times (0, 0.5, 1, 2, and 4 h) and analyzed LRP-1 mRNA and protein expression by RT-PCR and western blotting. LPS induced a time-dependent decrease in LRP-1 mRNA between 0.5 and 4 h. Significant decreases were observed at 1, 2, and 4 h relative to controls (*P* < 0.05) (Figure 4a-4c). We next examined LRP-1 expression in LPS-stimulated BV2 cells after treatment with ATX at various concentrations ranging from 0 to 10 μM. ATX at concentrations ranging from 2.5 μM to 10 μM reversed the LPS-induced down-regulation of LRP-1 mRNA and protein expression relative to untreated control BV2 cells in a dose-dependent manner (*P* < 0.05) (Figure 4d-4f).

### The LRP-1 antagonist receptor-associated protein inhibits M2 polarization by activating NF-κB and JNK signaling

**Figure 5:**
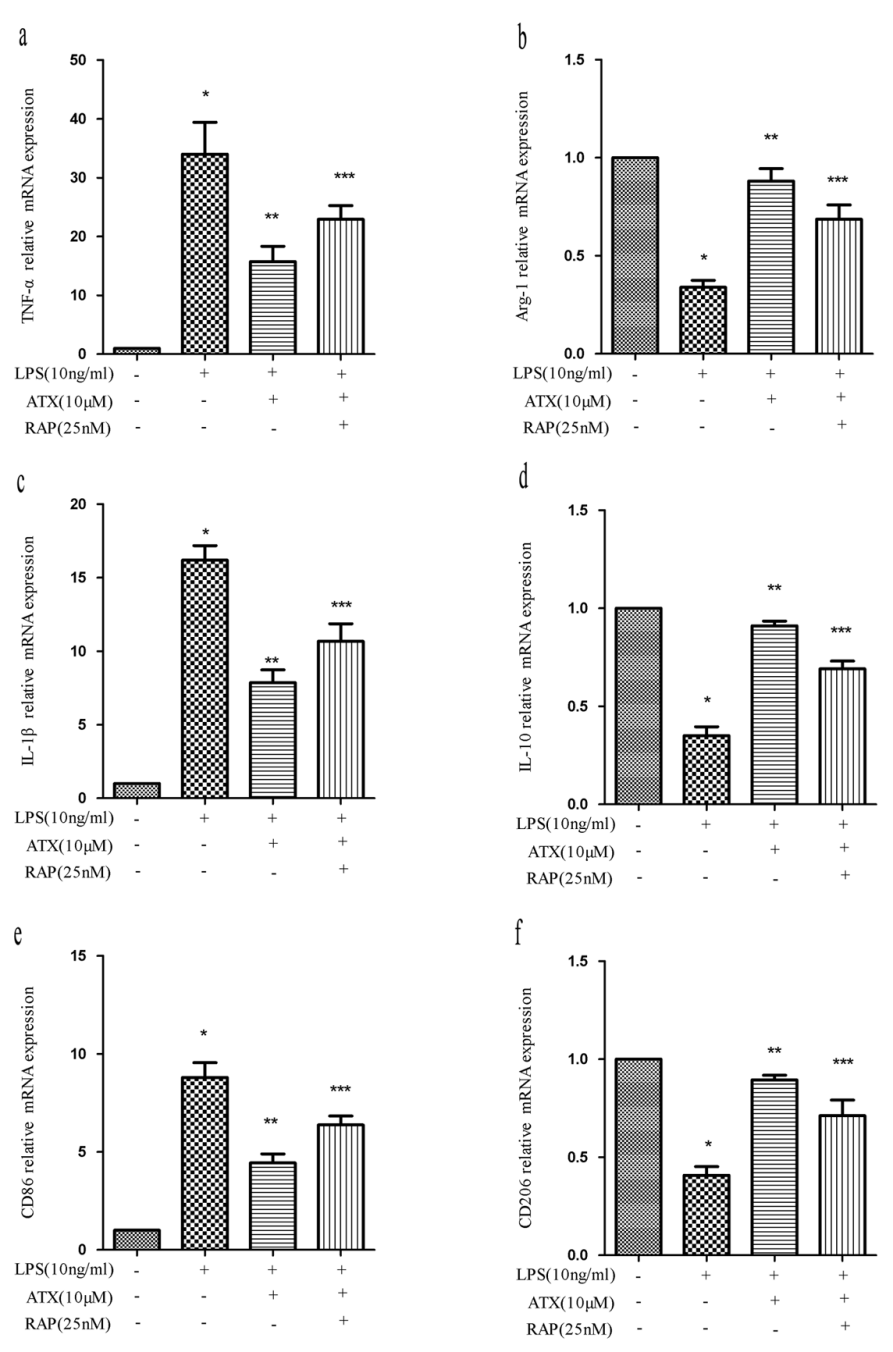
RAP inhibits ATX-induced M2 microglia polarization BV2 cells were incubated with ATX (10 μM) or RAP (25 nM) for 4 h prior to treatment with LPS (10 ng/mL) for 4 h. Total RNA was extracted and the expression of M1 (TNF-α, IL-1β, and CD86) and M2 (Arg-1, IL-10, and CD206) markers analyzed by RT-PCR. Fold changes in gene expression were calculated relative to β-actin. **a.**, **c.**, **e.** M1 marker expression. **b.**, **d.**, **f.** M2 marker expression. The data are presented as the mean ± SD (*n* = 3). ^*^P < 0.05 indicates a significant difference in the expression of the different markers compared to LPS (−)/ATX (−)/RAP (−)-treated cells. ^**^P < 0.05 indicates a significant difference compared to LPS (+)/ATX (−)/RAP (−)-treated cells. ^***^P < 0.05 indicates a significant difference compared to LPS (+)/ATX (+) /RAP (−)-treated cells.

**Figure 6:**
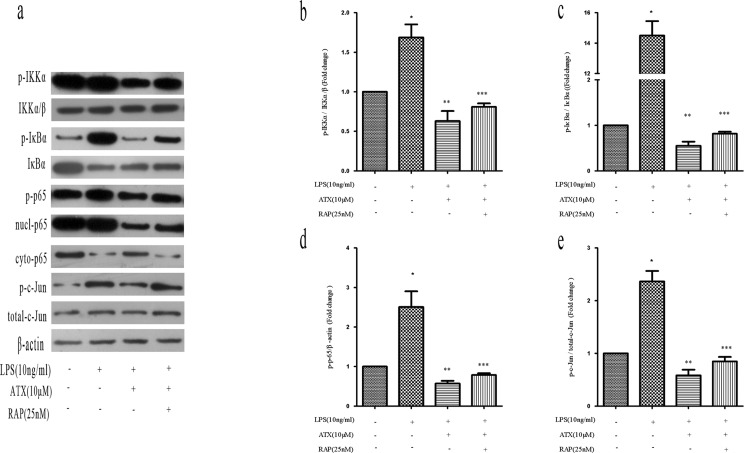
RAP inhibits ATX-induced M2 microglia polarization by promoting NF-κB and JNK activation BV2 cells were incubated with ATX (10 μM) or RAP (25 nM) for 4 h prior to treatment with LPS (10 ng/mL) for 4 h. **a.** Analysis of NF-κB and JNK signaling pathway proteins by western blotting. **b.** Analysis of the ratio of p-IKKα/p-IKKα/β expression. **c.** Analysis of the ratio of p-IκBα/IκBα expression. **d.** Analysis of the ratio of p-p65/β-actin expression. **e.** Analysis of the ratio of p-c-Jun/total c-Jun expression. Data are presented as the mean ± SD (*n* = 3). ^*^P < 0.05 indicates a significant difference in the expression of the different markers compared to LPS (−)/ATX (−)/RAP (−)-treated cells. ^**^P < 0.05 indicates a significant difference compared to LPS (+)/ATX (−)/RAP (−)-treated cells. ^***^P < 0.05 indicates a significant difference compared to LPS (+)/ATX (+) /RAP (−)-treated cells.

Receptor-associated protein (RAP) is a specialized endoplasmic reticulum (ER) chaperone for LDL receptor family members including LRP-1 [27], which binds to LRP-1 with high affinity and has been widely used as an antagonist of LRP-1 function [28]. To investigate whether RAP regulated ATX-induced M2 microglia polarization, we analyzed the expression of M1 (TNF-α, IL-1β, and CD86) and M2 (Arg-1, IL-10, and CD206) markers in BV2 cells. Pretreatment with RAP (25 nM for 30 min) resulted in an increase in the expression of M1 markers indicating RAP promoted M1 polarization (Figure 5a, 5c, 5e). Conversely, RAP reduced the ATX-enhanced expression of M2 markers in BV2 cells (Figure 5b, 5d, 5f), indicating RAP inhibited M2 polarization. We examined the effects of RAP on NF-κB and JNK signaling. Upon treatment with LPS and ATX, we observed decreased phosphorylation of IKKα, IκBα, and p65. In contrast, enhanced phosphorylation of IKKα, IκBα, and p-65 was observed upon addition of RAP, indicative of activation of NF-κB signaling (Figure 6a-6d). RAP induced an increase in p-c-Jun, which was indicative of activation of JNK signaling (Figure 6a, 6e). These results indicated that RAP inhibited ATX-induced M2 microglia polarization by activating NF-κB and JNK signaling.

### Inhibition of NF-κB and JNK signaling attenuates ATX-induced M2 polarization

**Figure 7:**
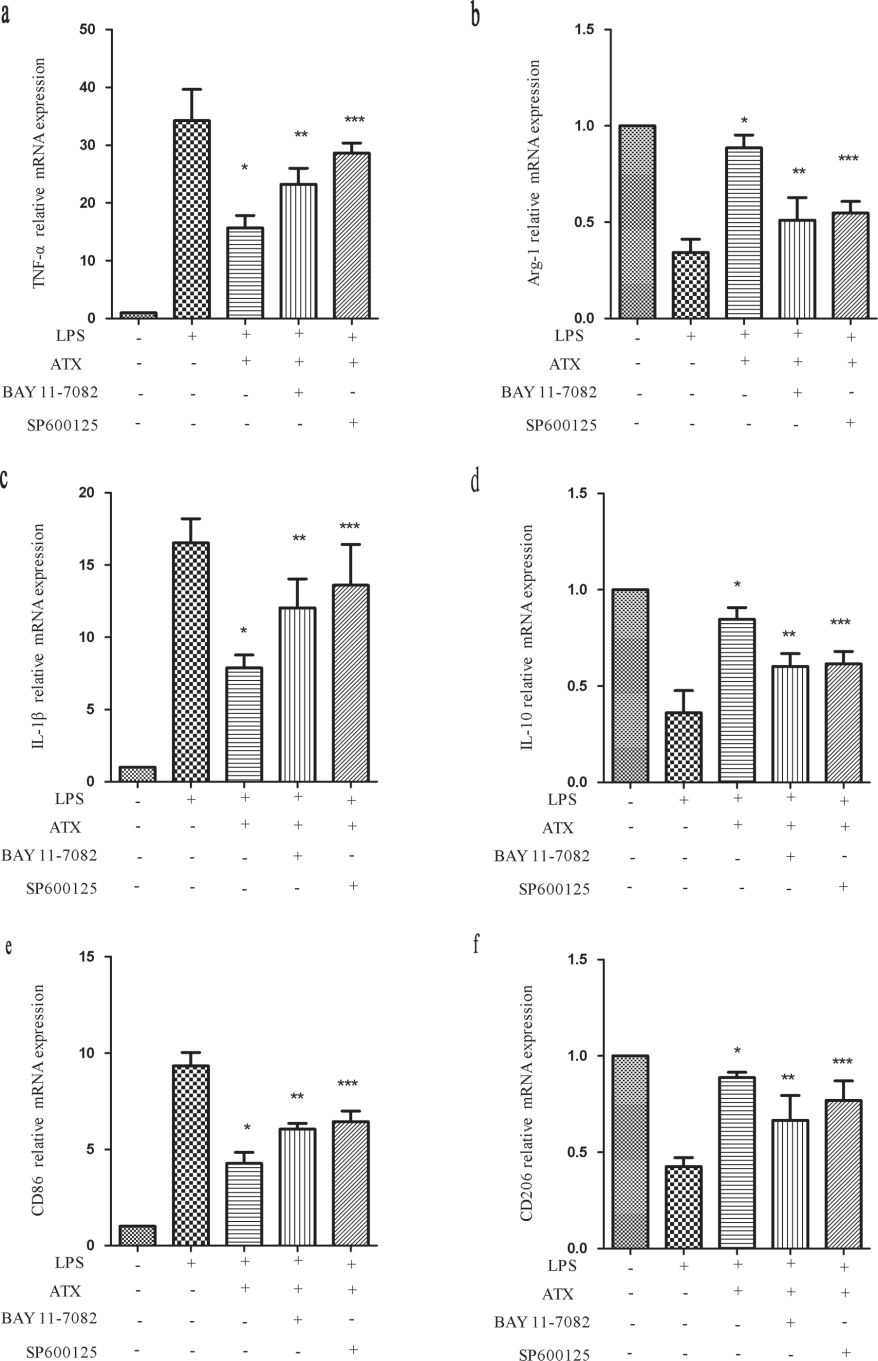
Inhibition of NF-κB and JNK signaling attenuates ATX-induced M2 microglia polarization BV2 cells were incubated with ATX (10 μM for 4 h), SP600125 (10 μM for 30 min), or Bay11-7082 (10 μM, for 30 min) prior to LPS treatment (10 ng/mL for 4 h). Total RNA was extracted and the expression of M1 (TNF-α, IL-1β, and CD86) and M2 (Arg-1, IL-10, and CD206) markers analyzed by RT-PCR. Fold changes in gene expression were quantified relative to β-actin. **a.**, **c.**, **e.** M1 marker expression. **b.**, **d.**, **f.** M2 marker expression. The data are presented as the mean ± SD (*n* = 3). ^*^P < 0.05 indicates a significant difference compared to LPS (+)/ATX (−)/SP600125 (−)/Bay11-7082 (−)-treated cells. ^**^P < 0.05 indicates a significant difference compared to LPS (+)/ATX (+)/SP600125 (−)/Bay11-7082(−)-treated cells. ^***^P < 0.05 indicates a significant difference compared to LPS (+)/ATX (+)/SP600125 (−)/Bay 11-7082 (−)-treated cells.

To confirm that ATX suppressed the proinflammatory response, we inhibited NF-κB activity with BAY11-7082 and JNK activity with SP600125. We examined the expression of M1 (TNF-α, IL-1β, and CD86) and M2 (Arg-1, IL-10, and CD206) markers after treatment with BAY11-7082, which inhibits NF-κB signaling by reducing p-IκBα levels [29]. Pretreatment of BV2 cells with ATX resulted in a decrease in TNF-α mRNA. However, this effect was abolished by pretreatment with BAY11-7082 (10 μM for 30 min). A similar trend was observed in IL-1β and CD86 mRNA expression (Figure 7a, 7c, 7e) compared to LPS- and ATX-treated cells (*P* < 0.05). ATX pretreatment reversed the reduction in Arg-1 mRNA expression. Pretreatment with an NF-κB inhibitor increased Arg-1 mRNA expression compared to control cells. Similar effects were observed in IL-10 and CD206 mRNA expression (Figure 7b, 7d, 7f). SP600125 is a cell-permeable small molecule that selectively inhibits JNK isoforms and prevents phosphorylation of c-Jun [30]. SP600125 treatment (10 μM for 30min) increased TNF-α, IL-1β, and CD86 mRNA levels (Figure 7a, 7c, 7e), and reduced the levels of Arg-1, IL-10, and CD206, which were elevated in response to ATX pretreatment (Figure 7b, 7d, 7f). Inhibition of NF-κB and JNK abolished M1 but enhanced M2 marker expression. Thus, ATX regulated M2 microglia polarization by inhibiting NF-κB and JNK signaling pathway activation.

## DISCUSSION

We investigated whether ATX reversed the M1/M2 polarization of LPS-induced BV2 cells to suppress neuroinflammation through LRP-1. Pretreatment with ATX promoted induced a neuroprotective phenotype by inhibiting proinflammatory M1 and promoting anti-inflammatory M2 polarization in LPS-stimulated BV2 microglial cells. We also elucidated the role LRP-1-dependent NF-κB and JNK inhibition in ATX-induced M2 polarization. Our data suggest that ATX promotes M2 polarization to suppress neuroinflammation in an LRP-1-dependent manner by modulating NF-κB and JNK signaling.

M1 and M2 microglia function is regulated by cytokines and microbial-derived products including LPS [31]. In an *in vitro* microenvironment, LPS and IFNγ promote M1 polarization, while IL-4 and IL-13 promote M2 polarization (Figure 1) [4]. M1 microglia release proinflammatory cytokines such as TNF-α, IL-6, and IL-1β to induce an inflammatory response [32]. In contrast, M2 microglia produce Arg1, IL-10, and neurotrophic factors such as nerve growth factor, which attenuate the inflammatory response [33]. The phenotypes are distinguished by specific markers [34]. We found that ATX pretreatment of LPS-treated BV2 cells resulted in down-regulation of M1 (TNF-α, IL-1β, and CD86) and enhanced expression of M2 (Arg-1, IL-10, and CD206) markers (Figure 1-2). The neuroprotective effects of ATX resulted, at least in part, from M2 polarization. Treatment of amyloid precursor protein (APP)/PS1 transgenic mice with the small molecule MW-151 attenuated microglial activation and the production of proinflammatory cytokines in the cortex. This resulted in protection from synaptic dysfunction, which has been implicated in learning and memory [35]. Therefore, it might be clinically beneficial to manipulate microglia phenotypes from M1 cytotoxic to M2 neuroprotective through drug treatment or genetic modification.

NF-κB activation is responsible for M1 macrophage polarization and inflammation in response to LPS in BV2 cells. Under normal conditions, the heterodimeric NF-κB subunits p50 and p65 form a complex with the intrinsic inhibitor IκBα in plasma. Phosphorylation of IκB by IKKα leads IκBα degradation, which allows NF-κB nuclear translocation and activation. NF-κB transcriptional activity is regulated by posttranslational modifications such as phosphorylation of p65 [36]. We found that ATX inhibited LPS-induced p65 phosphorylation and nuclear translocation in BV2 cells. The LPS-enhanced levels of p-IκBα and p-IKKα were reduced after pretreatment with ATX (Figure 3a, 3c). These results indicated that ATX reversed LPS-induced microglia activation through inhibition of NF-κB and JNK signaling. JNK signaling has a central role in neuroinflammation and contributes to AD. LPS was shown to induce JNK activation in normal microglia, which led to increased production of inflammatory cytokines [14]. We also observed aberrant JNK activation in LPS-induced BV2 cells, which was reversed by pretreatment with ATX (Figure 3a, 3c).

Because both the JNK and NF-κB pathways were responsible for microglial activation in LPS-stimulated BV2 cells, we investigated whether inhibition of these pathways could reverse ATX-induced M1/M2 polarization of microglia. Inhibition of the NF-κB and JNK pathways with BAY11-7082 and SP600125 revealed their intrinsic effects on M1/M2 polarization in BV2 cells (Figure 7a-7f). ATX inhibited the activation of NF-κB and JNK signaling to suppress neuroinflammation.

Microglial cells in brain tissue from wild-type mice expressed higher levels of LRP-1 [37]. Previous studies have suggested that LRP-1 regulates inflammation and has a protective role in microglia in human adults [38]. Reduced LRP-1 expression and increased levels of proinflammatory chemokines may act synergistically to promote microglial activation [39-40]. Chuang et al. demonstrated that in the brains of microglial *LRP-1* conditional knockout mice, microglia adopted a proinflammatory phenotype characterized by amoeboid morphology, indicating that LRP-1 may regulate microglial activation [41]. We observed aberrant down-regulation of LRP-1 mRNA and protein levels in LPS-induced BV2 cells, which was reversed by ATX in a dose-dependent manner (Figure 4). These results indicate LRP-1 has an important role in ATX-induced M2 polarization. Additional studies are needed to understand the mechanisms underlying ATX-mediated LRP-1 activation in microglia.

The production of cytokines such as TNF-α, IL-6, and IL-1β, is tightly controlled by NF-κB signaling. NF-κB is a master transcriptional regulator of the inflammatory response [42]. Blocking NF-kB activation specifically in microglia has a neuroprotective effect during experimental autoimmune encephalitis [41]. LRP-1 can inhibit inflammation through regulation of NF-κB activity [40]. Additionally, Yang et al. demonstrated that suppression of LRP-1 results in activation of NF-κB and JNK signaling, suggesting that LRP-1 directly regulates signaling pathways that are critical for the inflammatory response in microglial cells [15]. We found that RAP induced phosphorylation of IKKα, IκBα, and p-65 in BV2 cells, which was indicative of NF-κB activation. Consistent with the results of Chuang et al. [41], we demonstrated that RAP promoted NF-κB activation in the presence of inflammatory stimuli. Therefore, we speculated that the interaction between LRP-1 and NF-κB could be important for ATX-mediated inhibition of NF-κB activation. Although a previous study suggested that LRP-1 inhibited NF-κB activation in a MyD88-dependent manner [41], the underlying mechanisms have not been elucidated.

LPS can promote activation of MAPK signaling pathways [43]. Pocivavsek et al. demonstrated that LDL receptor activation reversed glial inflammation *via* JNK activation, and that the anti-inflammatory effects of an apoE mimetic peptide reduced JNK activity [44]. We determined that RAP promoted phosphorylation of c-Jun, consistent with a previous study [15]. These findings support the hypothesis that LRP-1 mediated the anti-inflammatory effects of ATX in LPS-induced BV2 cells. Interactions between the cytoplasmic domains of LRP-1 (LRP-1-ICD) and JNK-interacting proteins regulate JNK signaling [45]. Overexpression of the LRP-1-ICD selectively inhibited activated JNK from translocating into the nucleus, and reduced phosphorylation of c-Jun [46]. Indeed, an LRP-1 agonist attenuated the expression of proinflammatory mediators, while an antagonist had the opposite effect in macrophages [47].

LRP-1 participates in a variety of pathways linked to AD pathogenesis. It regulates endocytosis of ligands including ApoE and Aβ. It also mediates Aβ clearance through cellular uptake followed by lysosomal degradation and/or transcytosis across the blood brain barrier to the circulation and subsequent peripheral clearance [48-49]. Binding of APP to LRP-1 resulted in increased clearance of APP [50] and deletion of LRP-1 exacerbated Aβ deposition [10]. Dysregulation of LRP-1 has been associated with the aberrant activation or impaired function of the innate immune system in AD. Additionally, the glial activation observed in AD brains may represent a failure of LRP-1 immunomodulatory functions in the presence of chronic insults by Aβ oligomers [51]. Given that LRP-1 functions in both inflammation and Aβ clearance, it may have a key regulatory role in AD.

**Figure 8:**
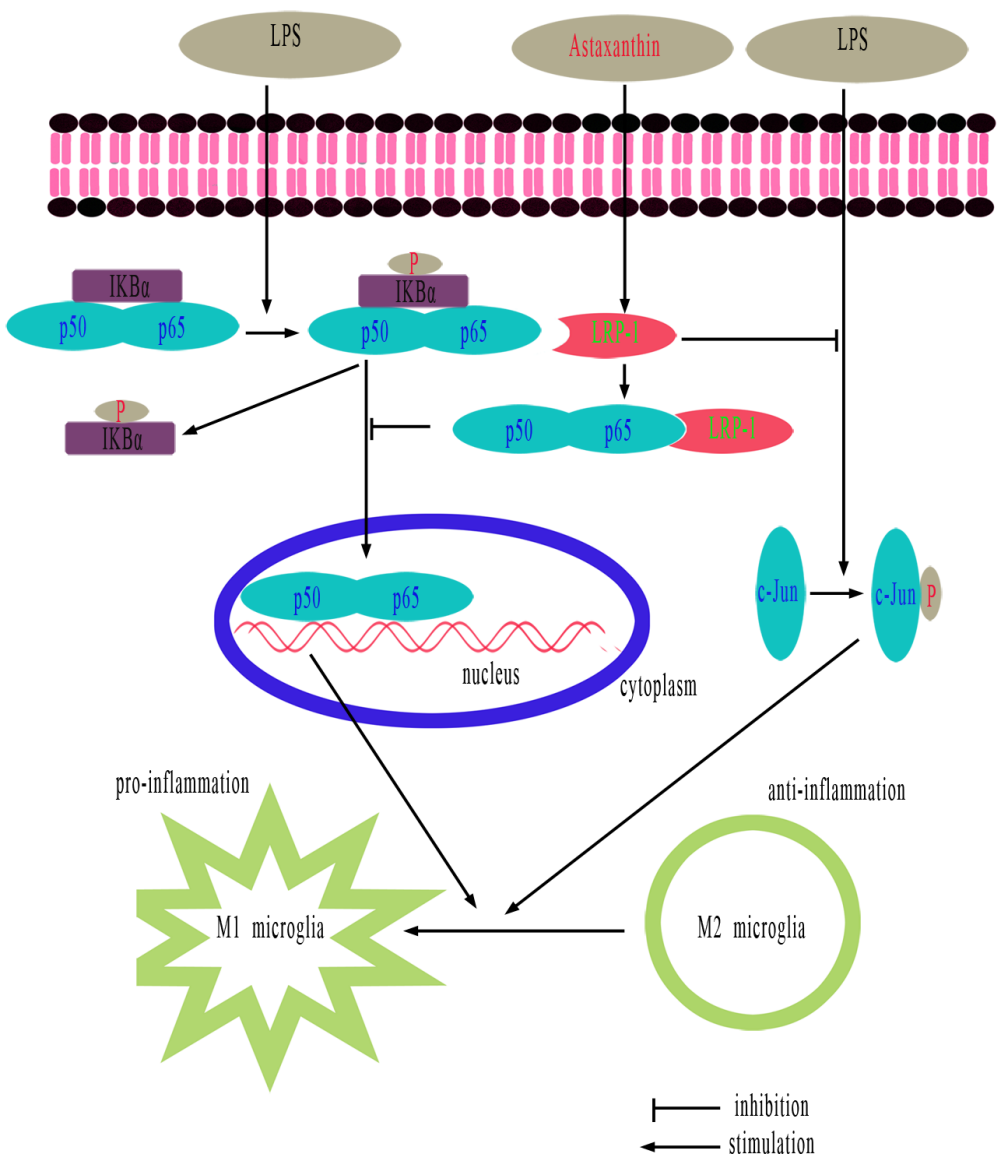
Schematic showing the proposed anti-inflammatory effects of ATX ATX promotes M2 and inhibits M1 polarization in LPS-stimulated BV2 cells by attenuating JNK and NF-κB pathway activation and inflammation through LRP-1. LRP-1 could either directly or indirectly mediate inhibition of inflammatory signaling pathways following LPS stimulation.

In summary, ATX elevates LRP-1, which can bind and down-regulate both NF-κB p65 and p-c-Jun, thus preventing the activation of the NF-κB and JNK pathways. The repression of NF-κB and JNK activation reverses LPS-induced M1 microglia polarization, thereby attenuating the neuroinflammatory response. Restoration of LRP-1 expression in microglia may serve as a novel therapeutic approach to combat microglial dysfunction associated with chronic inflammation in neurodegenerative diseases including AD. This study provides a molecular basis for further studies of ATX in LPS-induced microglia inflammatory responses in BV2 cells (Figure 8). ATX promotes M2 microglia polarization to suppress neuroinflammation in an LRP-1-dependent manner by inhibiting NF-κB and JNK activation. Therefore, ATX may be an effective therapeutic for AD.

## MATERIALS AND METHODS

### Chemicals

ATX was purchased from Sigma-Aldrich (St. Louis, MO, USA). Recombinant mouse RAP was purchased from R&D Systems (Minneapolis, MN, USA). The following primary antibodies were used for western blotting: mouse/rabbit anti-p-IKKα, anti-IKKα/β, anti-p-IκB, anti-IκB, anti-p-p65, anti-p65, anti-p-c-Jun, anti-total-c-Jun, and anti-β-actin. All antibodies were purchased from Cell Signaling Technology (Danvers, MA, USA). LPS, the NF-κB inhibitor (BAY 11-7082), and JNK inhibitor (SP600125) were purchased from Sigma-Aldrich. The Nuclear and Cytoplasmic Protein Extraction Reagents and BCA protein assay kit were obtained from Beyotime Biotechnology (Haimen, China). SYBR RT-PCR Kits were purchased from Takara Shuzo (Shiga, Japan).

### Cell culture

BV2 cells are an immortalized murine microglial cell line, which is frequently used as a substitute for primary microglia [52]. BV2 cells were cultured in DMEM (HyClone, Carlsbad, CA, USA) supplemented with 10% fetal bovine serum (Gibco, Carlsbad, CA, USA), 100 U/mL penicillin, and 100 ug/mL streptomycin at 37°C in a humidified atmosphere containing 5% CO_2_ and 95% air. Cells were pretreated with or without ATX (0 to 10μM) for 4 h and then co-incubated with LPS 10 ng/mL for 4 h to evaluate the protective effects of ATX.

### ELISA

BV2 cells were incubated with ATX in the indicated concentrations (0, 2.5, 5, and 10 μM) for 4 h. LPS (10 ng/mL) was then added and the cells incubated for 4 h. TNF-α and IL-10 production were analyzed in BV2 cell conditioned media 24 h after incubation using an ELISA kit (Dakewe, Shenzhen, China). The levels of TNF-α and IL-10 were quantified using the manufacturer’s protocol.

### Quantitative RT-PCR

BV2 cells were seeded into six-well plates and treated as indicated. Total RNA was extracted using the Trizol reagent according to the manufacturer’s instructions. RT-PCR were performed using the One-Step RT-PCR System (Applied Biosystems, Foster City, CA, USA). One microgram of RNA template was reverse-transcribed using a Prime Script RT Master Mix Kit (Clontech, Mountain View, CA, USA). RT-PCR was performed using 2 μL of cDNA solution in a 20 μL reaction mixture containing 10 μL of SYBR Premix Ex Taq II, 0.8 μL of the forward primer, 0.8 μL of the reverse primer, and 6 μL ddH_2_O. Relative mRNA expression was assessed using the comparative ^ΔΔ^Ct method. Β-actin was used as an internal standard. The RT-PCR primers are shown in Table 1.

**Table 1:** Primers for RT-PCR.

Gene	Forward(5′-3′)	Reverse (5′-3′)
LRP-1	ACTATGGATGCCCCTAAAACTTG	GCAATCTCTTTCACCGTCACA
TNF-α	CATCTTCTCAAAATTCGAGTGAC	TGGGAGTAGACAAGGTACAACCC
Arg-1	CGCCT TTCTCAAAAGGACAG	CCAGCTCTT CATTG G C TTTC
IL-1β	TGTGCAAGTGTCTGAAGCAGC	TGGAAGCAGCCCTTCATCTT
IL-10	GCTCTTACTGACTGGCATGAG′	CGCAGCTCTAGGAGCATGTG
CD206	CTTCGGGCCTTTGGAATAAT	TAGAAGAGCCCTTGGGTTGA
CD86	ACGATGGACCCCAGATGCACCA	GCGTCTCCACGGAAACAGCA
β-actin	GG CATC GT G ATGGACTC CG	GCTG GAAGGT GGA CAGCGA

### Preparation of nuclear and cytosolic fractions

Cells were homogenized in PER-Mammalian Protein Extraction Buffer (Pierce Biotechnology, Rockford, IL, USA) containing protease inhibitor cocktail I and 1 mM phenylmethylsulfonyl fluoride. Cytosolic fractions were prepared by centrifugation at 15,000 × g for 10 min at 4°C. Nuclear and cytoplasmic extracts from BV2 cells were prepared using the NE-PER nuclear and cytoplasmic extraction reagents (Pierce Biotechnology).

### Western blotting

BV2 cells were seeded onto six-well plates and treated as indicated. The cells were then harvested and lysates centrifuged at 12,000 × g for 15 min at 4°C. Supernatants was collected and total protein levels measured using the BCA protein assay kit (Beyotime Biotechnology). Equal amounts of total protein were separated by SDS-PAGE and transferred to PVDF membranes (Millipore). The membranes were incubated at room temperature for 1 h in blocking buffer and then incubated with the primary rabbit monoclonal antibodies (Cell Signaling Technology). Finally, the membranes were incubated with HRP-linked secondary antibodies and blots developed using the ECL reagent. After exposure to X-ray film, protein bands were imaged using a UV imaging system. Band intensities were quantified using the ImageJ software. β-actin was used as a loading control.

### Statistical analysis

All data are expressed as the mean ± standard deviation (SD). Statistical analyses were performed using SPSS version 17.0 (SPSS Inc., Chicago, IL, USA). Multiple groups were compared using analysis of variance followed by post-hoc Fisher’s least significant difference testing when appropriate. A *P* < 0.05 was considered significant.

## Abbreviations

ATX: Astaxanthin
AD: Alzheimer’s disease
Aβ: β-amyloid
IL-1β: Interleukin-1β
JNK: c-Jun N-terminal kinase
LPS: Lipopolysaccharide
LRP-1: Low-density lipoprotein receptor-related protein 1
NF-κB: Nuclear factor-κB
RT-PCR: Real-time PCR
RAP: Receptor-associated protein
TNF-α: Tumor necrosis factor-α
ELISA: Enzyme-linked immunosorbent assay
